# Does Prospective Mental Imagery Predict Symptoms of Negative Affect and Anhedonia in Young People?

**DOI:** 10.1007/s10578-024-01695-1

**Published:** 2024-05-05

**Authors:** Taryn Hutchinson, Laura Riddleston, Iris Lavi, Victoria Pile, Alan Meehan, Meenakshi Shukla, Jennifer Lau

**Affiliations:** 1 Department of Psychology, Institute of Psychiatry, Psychology & Neuroscience, King’s College London, London, UK; 2 Department of Psychology, University of Bath, Bath, UK; 3 School of Social Work, The Centre for Research and Study of the Family, University of Haifa, Haifa, Israel; 4 Department of Psychology, University of Allahabad, Prayagraj, India; 5 Youth Resilience Unit, Queen Mary, University of London, London, UK; 6 Newham Centre for Mental Health, London, E13 8SP UK

**Keywords:** Adolescence, Depression, Negative Affect, Anhedonia, Mental imagery

## Abstract

Adolescent depression is associated with unhelpful emotional mental imagery. Here, we investigated whether vividness of negative and positive prospective mental imagery predict negative affect and anhedonia in adolescents. 111 people from Israel completed measures of prospective mental imagery, negative affect, and anhedonia at two time-points approximately three months apart. Using three cross-lagged panel models, we showed once ‘concurrent’ (across-variable, within-time) and ‘stability’ paths (across-time, within-variable) were estimated, there were no significant cross-lag paths between: i) T1 prospective negative mental imagery and T8 negative affect (i.e. increased vividness of negative future imagery at Time 1 did not predict increased negative affect at Time 8); ii) T1 prospective positive mental imagery and T8 negative affect (i.e. reduced vividness of positive future imagery at Time 1 did not predict increased negative affect at Time 8); and iii) T1 prospective positive mental imagery and T8 anhedonia (i.e. reduced vividness of positive future imagery at Time 1 did not predict increased anhedonia at Time 8). Given high levels of attrition, future research should aim to explore these associations in a larger, more diverse population, as such data could inform on whether modifying earlier prospective mental imagery may influence later time/context-specific effects of prospective mental imagery on negative affect and anhedonia.

## Introduction

Depression is common, affecting approximately 280 million people worldwide (Institute of Health Metrics and Evaluation [IHME] [22], as cited by World Health Organization [WHO], [44]), and serious; being a leading cause of global disability [44]. Depression often starts in adolescence, and it is estimated that, globally, 34% of adolescents aged between 10 and 19 years are at risk of developing depression at a clinical level [38]. Adolescent depression is associated with social dysfunction, academic difficulties) and, if untreated, recurrent episodes and increased risk of chronicity [12, 36], further contributing to the development of lifelong mental and physical health conditions in adulthood [41]. While cognitive-behavioural interventions are used as frontline treatments, they are rarely applied in *preventative* programmes to reduce adolescent depressive symptoms [45], as they can be resource-intensive and costly. In order to develop innovative cognitive-behavioural preventative programmes, we need to better understand the cognitive correlates and precursors of adolescent depression. Previous research [21] has drawn on the potential of future mental imagery, the ability to imagine future images vividly, as an intervention target in preventative programmes. Hutchinson et al. [21] investigated whether vividness of future mental imagery, both positive and negative, associated with two core symptoms of adolescent depression: negative affect and anhedonia (lack of positive affect). However, as these previous associations were limited by the study’s cross-sectional design, the present study aimed to explore the across-time associations using longitudinal data.

Mental imagery refers to the capacity to ‘see with the mind’s eye’, whereby mental representations of sensory information are constructed using internal representations (e.g. past memories and predictions of the future [16, 23, 32]). *Emotional* mental imagery refers to representations that are affective in nature or function. In adults, unhelpful emotional mental imagery, such as frequent, vivid, and distressing memories of negative life events, has been implicated in the maintenance of depression [5], as well as several other affective conditions such as social phobia [19] and post-traumatic stress disorder [18]. Such unwanted memories have been reported by 44–87% of adults with depression [2, 4, 5, 25], 30. In addition to the presence of frequent, vivid negative images, reduced vividness of positive mental imagery, especially of future events, has also been associated with depression in adults [27, 28, 40]. Similar results have been found in adolescent populations, whereby depressed adolescents have more vivid negative memories than adolescents who have never been depressed [24]. Looking more systematically at negative and positive imagery for past and future events, a cross-sectional study by Pile and Lau [34] reported that adolescent depression symptoms were associated with increased vividness of negative events (past and future) *and* reduced vividness of positive events (again, both past and future). These associations were in contrast to associations with anxiety symptoms, which were only associated with vivid imagery of negative past events. We recently elaborated on these associations, again in adolescents, finding differential associations between future mental imagery variables and two key symptom dimensions of depression: negative affect and anhedonia. Our data showed that increased vividness of negative future imagery *and* reduced vividness of positive future imagery were associated with increased negative affect, but only reduced vividness of positive future imagery was associated with increased symptoms of anhedonia [21].Please confirm if the inserted city and country name of Affiliation 3 is correct. Amend if necessary.This is correct, thank you

These findings are important because they have implications for developing new psychological preventative interventions. Specifically, they suggest that enhancing positive affect through generation of imagery of positive future events, along with reducing the emotional impact of negative imagery, may be a promising mechanism to reduce not only negative affect but also anhedonia in depression [37]. Indeed, a growing body of literature shows that targeting distressing mental imagery using imagery re-scripting reduces depression in adults [4, 43] and adolescents [33], and that increasing the vividness of positive future mental imagery reduces symptoms of anhedonia [3] and increases positive affect [15] in adults with depression. However, these treatment implications may be supported further by establishing a cross-time association between mental imagery variables and depressive symptoms. As such data is currently lacking, the current study aimed to establish a directional relationship between negative and positive future imagery and negative affect and anhedonia using longitudinal data.

Drawing on the cross-sectional findings by Hutchinson et al., [21], we made three hypotheses: (i) that increased vividness of negative future imagery at baseline would be associated with greater negative affect at follow-up, (ii) reduced vividness of positive future imagery at baseline would also be associated with greater negative affect at follow-up, and (iii) reduced vividness of positive future imagery at baseline would be associated with increased anhedonia at follow-up. We used data gathered from young people during the lockdown phases of the COVID-19 pandemic to test our hypotheses. As the pandemic presented young people with various challenges, exploring depressive symptoms (negative affect, anhedonia) as a function of mental imagery variables offers a lens for understanding individual differences at this juncture. Data were from two time-points across the first wave of lockdowns as experienced by young people in Israel. Given the first-time use of the Prospective Imagery Task (PIT) in adolescents from Israel, we also aimed to assess its factor structure, along with age and sex differences. Further understanding the specific relationships between mental imagery and negative and positive affect may bridge the gap in preventative programmes for adolescent depression.

## Methods

### Materials and Methods

#### Participants and Procedures

Participants were 268 young people aged 12–18 years (mean = 15.41 years; SD = 1.77; 51.9% male; 48.1% female) recruited online via an Israeli survey company (iPanel). Participants who were signed up to iPanel could choose to take part in this study and were directed to a link to the study if they chose to do so. The inclusion criteria for participation was anyone between the ages of 12 and 18 years, residing in Israel at the time of data collection. The exclusion criteria was an inability to read and understand the study materials. The study received ethical approval from the Psychiatry, Nursing and Midwifery Research Ethics Committee at King’s College London (ref: HR-19/20-18250), the University of Haifa (ref: 368/20) and the University of Bath (ref: 4688 20-05469). A repeated online survey design was used to understand how young people in Israel were managing their emotions throughout the COVID-19 pandemic. Informed consent was provided by participants aged 18 years and parental consent was sought for participants between 12 and 17 years. Once parents gave consent for their child’s participation, participants were presented with a young person information sheet and asked for their assent. Only participants under 18 years who had full parental consent and their own assent were able to complete the survey questions.

Data was collected using the online platform Qualtrics between 17th May 2020 and 13th September 2020. Participants were given the opportunity to complete up to 8 surveys, which were sent out every two weeks for up to 16 weeks. Participants were reimbursed with 5 shekels (1.5 USD equivalent) per survey for their time. At baseline, 491 responses were recorded and 223 were removed due to having no data. Of the 268 young people with data at baseline, only 111 responses (41%) were recorded for the last survey (Time 8). Participants who completed surveys at Time 1 and Time 8 (completers), did not significantly differ from participants who only completed the Time 1 survey (T1 only), in terms of negative affect (completers: *M* = 20.97, *SD* = 8.12; T1 only: *M* = 20.84, *SD* = 8.61, *t*(266) = − 0.13, *p* = 0.899), anhedonia (completers: *M* = 30.06, *SD* = 4.78; T1 only *M* = 28.94, *SD* = 5.28, *t*(266) = − 1.79, *p* = 0.074), positive future imagery (completers: M = 25.65, *SD* = 5.78; T1 only: *M* = 25.72, *SD* = 2.81, *t*(261) = 0.10, *p* = 0.922) or negative future imagery (completers: M = 20.64, *SD* = 5.87; T1 only: *M* = 21.51, *SD* = 5.79, *t*(261) = 1.20, *p* = 0.231). There were no significant differences in sex between completers and those who completed Time 1 only, χ^2^ (1, *N* = 268) = 2.31, *p* = 0.128. There were also no significant differences in age between completers (*M* = 15.33, *SD* = 1.77) and those who completed Time 1 only (*M* = 15.45, *SD* = 1.77), *t*(266) = 0.48, *p* = 0.315. Survey questions covered demographics, school attendance, the impact of the COVID-19 pandemic on different life domains, as well as self-reported primary outcomes (measures of negative affect, anhedonia, well-being, loneliness, and boredom) and predictors of these outcomes (prospective mental imagery, attentional control, and appraisal abilities). Participants were also asked open-ended questions about the content of their worries, and ways they managed negative emotions and loneliness. The present analysis focuses on negative affect, anhedonia and prospective mental imagery at Time 1 (T1) and Time 8 (T8), approximately 3 months apart (mean days = 103.10, SD = 6.69, range = 97 and 119). These time points were chosen to capture more persistent associations over time.

#### Measures

##### Demographic Characteristics

Participants provided their sex assigned at birth and the month and year of birth.

##### Positive and Negative Affect Schedule (PANAS)

The Negative subscale of the PANAS (PANAS-N; [42]) was used to measure negative affect. The PANAS-N consists of 10 items rated on a 5-point Likert scale, with higher scores indicating greater negative affect. The PANAS-N demonstrated excellent internal consistency at Time 1 (Cronbach’s α = 0.90) and Time 8 (Cronbach’s α = 0.91). A previous study of young adults and adolescents translated the PANAS into Hebrew and found that the PANAS-N had good internal consistency (Cronbach’s α = 0.83 [1],). In non-Hebrew young people, the PANAS has also been shown to have convergent and discriminant validity with measures of anxiety, depression and well-being (Ortuño-Sierra et al. 2019, Sadin 2003). However, in our sample, the test re-test reliability was poor (*r* = 0.42, *p* < 0.001).

##### Snaith-Hamilton Pleasure Scale (SHAPS)

The SHAPS (based on [39]) is a measure of anhedonia comprising 14 items. This study used an adapted version of the SHAPS [21], for use in young people during the pandemic by removing five items that were less relevant during lockdown conditions). The remaining nine statements were presented to participants (e.g. “I would find pleasure in my hobbies and pastimes”) and participants indicated how much they agreed or disagreed with each statement on a 4-point Likert scale (Franken & Muris 2007). Higher scores indicate greater anhedonia. The internal consistency for the SHAPS was good at Time 1 (Cronbach’s α = 0.83) and excellent at Time 8 (Cronbach’s α = 0.91). These results are consistent with a previous large adolescent sample study [21], which found the SHAPS to have good internal consistency (Cronbach’s α = 0.87) albeit in a UK sample using the English language scale. Another study, also of English-speaking young people, found good convergent and discriminant validity (Leventhal et al., 2015). Here, the test re-test reliability for the SHAPS was poor (*r* = 0.50, *p* < 0.001).

##### The Prospective Imagery Task (PIT)

The PIT is a measure of vividness for prospective mental imagery and was originally used with adults [17], 40) but has been adapted for use in young people [34], 2020). The Hebrew translation of the PIT was developed through back-translation by one of the authors and a research assistant. Fourteen scenarios (7 positive and 7 negative) are presented to participants (“You will achieve something you wanted to” and “You will have a serious argument with a friend” as positive and negative examples, respectively) and they are asked to imagine each happening to them and then rate the vividness of this mental image on a 5-point scale (from ‘No image at all’ to ‘Very clear and detailed’). Higher scores indicate more vivid future images on the two scales. At Time 1, the positive scale demonstrated good internal consistency (Cronbach’s α = 0.80) and acceptable internal consistency was noted for the negative scale (α = 0.77). At Time 8, the positive scale demonstrated excellent internal consistency (Cronbach’s α = 0.90) and good internal consistency for the negative scale (Cronbach’s α = 0.86). These reliability statistics are broadly similar to a large UK based study of young people [21],positive scale (Cronbach’s α = 0.83) and negative scale (Cronbach’s α = 0.75). The test–retest reliability was poor for both the positive scale (*r* = 0.52, *p* < 0.001) and the negative scale (*r* = 0.50, *p* < 0.001).

### Data analysis

First, the 2-factor structure of the Prospective Imagery Task at Time 1 and Time 8 was confirmed using Principal Component Analysis (PCA) in IBM SPSS Statistics (Version 28). Next, we investigated the effects of key demographic factors (i.e. age and sex) on emotional mental imagery, and their associations with negative affect and anhedonia at both time points using independent samples t-tests and paired samples t-tests (sex) and correlations (age). Correlations (within and across time) between future mental imagery, negative affect, and anhedonia were also examined.

**Fig. 1:**
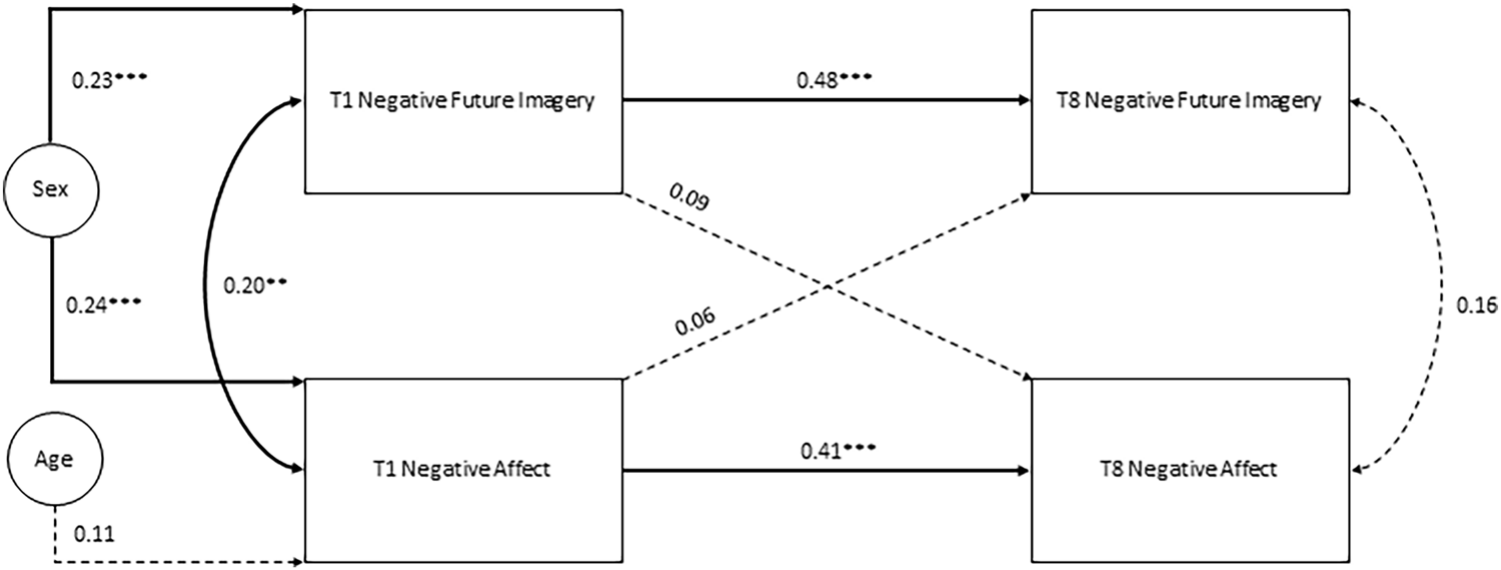
Cross-lagged panel model for negative future imagery and negative affect, testing within-time (concurrent) associations between variables, cross-time (stability) associations within variables, and cross-time, cross-variable (causal) associations. All associations control for the effects of sex on negative future imagery and negative affect at Time 1, and the effects of age on negative affect at Time 1. Significant paths are represented by solid lines. *p < .05, **p < .01, ***p < .001

**Fig. 2:**
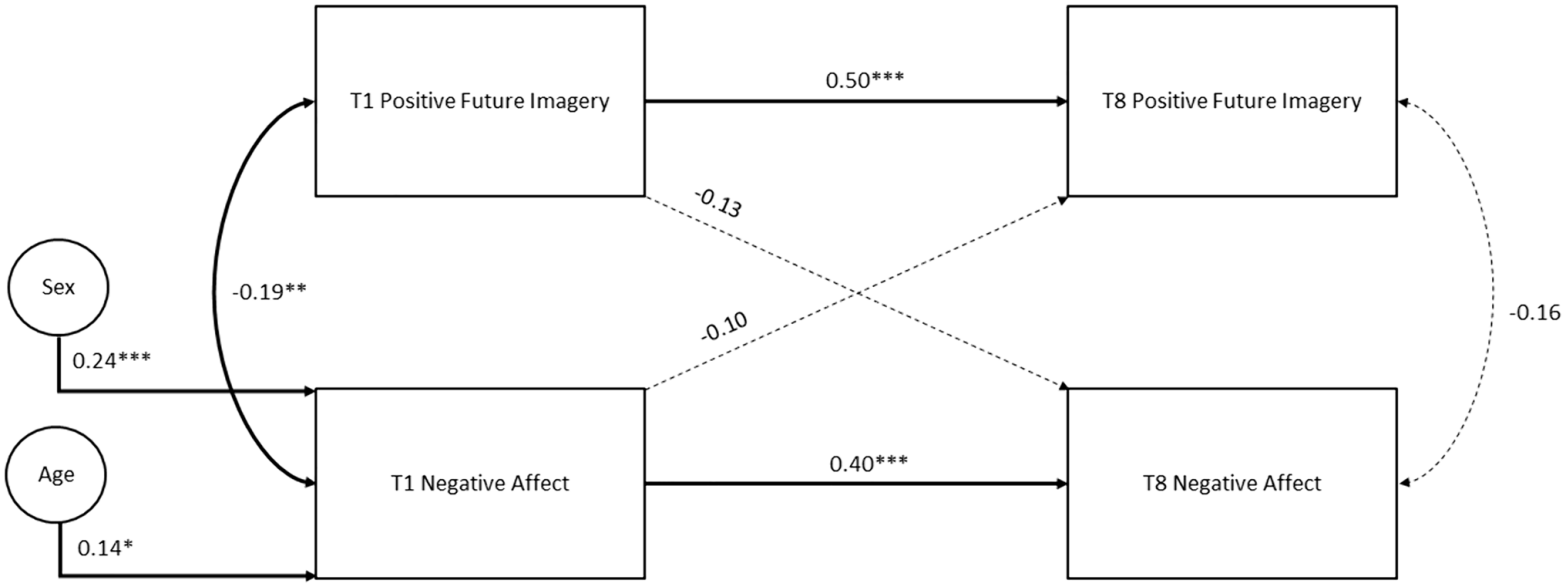
Cross-lagged panel model for positive future imagery and negative affect, testing within-time (concurrent) associations between variables, cross-time (stability) associations within variables, and cross-time, cross-variable (causal) associations. All associations control for the effects of sex and age on negative affect at Time 1. Significant paths are represented by solid lines. *p < .05, **p < .01, ***p < .001

**Fig. 3:**
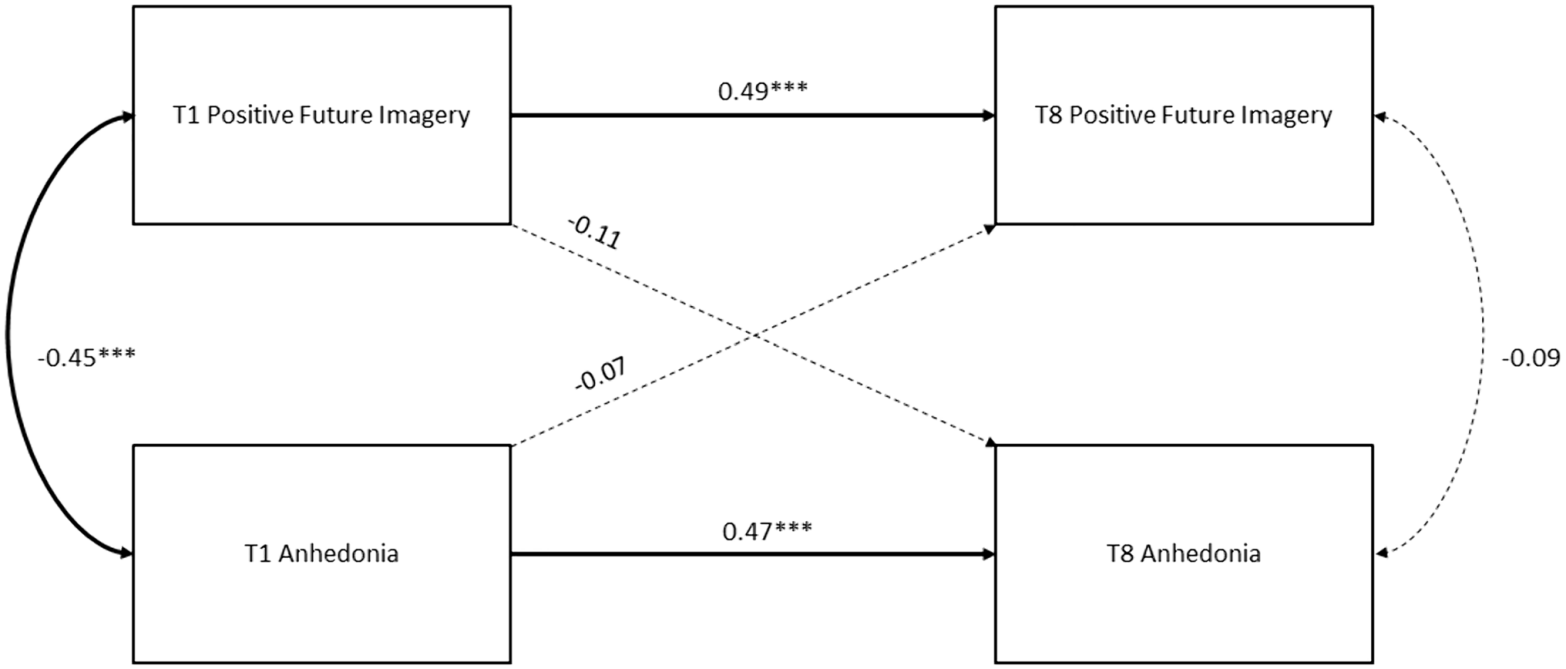
Cross-lagged panel model for positive future imagery and anhedonia, testing within-time (concurrent) associations between variables, cross-time (stability) associations within variables, and cross-time, cross-variable (causal) associations. Significant paths are represented by solid lines. *p < .05, **p < .01, ***p < .001

To test our three main hypotheses, we conducted two sets of analysis. First, multiple regression analyses were used to confirm that both negative and positive future imagery at Time 1 predicted negative affect at Time 8, but that only Time 1 positive future imagery predicted anhedonia at Time 8, as per previous findings by Hutchinson et al., [21]. Next, within more stringent cross-lagged panel models developed in MPlus v8 [29], we estimated path coefficients for (i) concurrent (i.e. within-time) associations between the different constructs, (ii) cross-time associations within the same construct (stability), and (iii) cross-time associations between different constructs (cross-lags consistent with ‘causal’ associations). Unlike typical regression analysis, cross-lagged designs enable simultaneous assessment of reciprocal paths between vividness of negative future imagery and negative affect (Fig. 1), vividness of positive future imagery and negative affect (Fig. 2), and vividness of positive future imagery and anhedonia (Fig. 3. Sex and age were included as covariates if they significantly associated with mental imagery and depressive symptom variables at any time-point. Model parameters were estimated using maximum likelihood estimation and full information maximum likelihood accounted for any missing data. Model fit was assessed using various goodness-of-fit measures (chi-square test, comparative fit index [CFI], Tucker-Lewis index [TLI] and root mean-squared error of approximation [RMSEA]. Non-significant chi-squares, CFI values ≥ 0.95, TLI values ≥ 0.95, and RMSEA values < 0.08 indicate good model fit [6, 20].

## Results

### Principal Component Analysis

A PCA [11] specifying two factors was run separately for Time 1 and Time 8 data. For Time 1 data, Component 1 explained 26% of the variance while Component 2 explained 19% of the variance. As shown in Table 1, the positive PIT items loaded onto the positive scale, while negative PIT items loaded onto the negative scale. Results were similar at Time 8, where two distinct constructs of negative and positive future mental imagery emerged. At Time 8, Component 1 explained 37% of the variance while Component 2 explained 23% of the variance.

**Table 1:** Factor loadings based on confirmatory factor analysis with orthogonal rotation for the 14 items of the Prospective Imagery Task (PIT) at Time 1 (N = 263) and Time 8 (N = 110)

	Time 1		Time 8	
	PIT-N	PIT-P	PIT-N	PIT-P
You will have a serious argument with a friend	**0.570**	0.281	**0.674**	.187
You will be unwell	**0.621**	0.117	**0.805**	.166
You will feel that people don’t understand you	**0.650**	0.098	**0.788**	.228
You will find your (school) work really difficult	**0.617**	− 0.043	**0.616**	.087
Things won’t work out as you had hoped	**0.686**	0.146	**0.765**	.052
People will find you dull and boring	**0.690**	− 0.144	**0.678**	− .024
People won’t like you	**0.661**	− 0.032	**0.778**	− .087
You will have lots of energy and enthusiasm	0.225	**0.588**	.049	**0.801**
You will do well at school/ work	− 0.048	**0.635**	.073	**0.770**
You will make good and lasting friendships	0.050	**0.639**	.032	**0.796**
You will achieve something you wanted to	− 0.020	**0.750**	.154	**0.800**
People you meet will like you	0.059	**0.759**	.106	**0.770**
You will be very fit and healthy	0.026	**0.640**	.079	**0.766**
You will have lots of fun with friends	0.090	**0.673**	.099	**0.812**

### Demographic Effects on Future Mental Imagery, Negative Affect and Anhedonia

Table 2 shows the means and standard deviations for negative affect, anhedonia, and positive and negative future imagery at Time 1 and Time 8. Female participants (*M* = 23.01, *SD* = 8.82) showed significantly higher negative affect than male participants (*M* = 18.94, *SD* = 7.48; *t*(266) = − 4.08*, p* < 0.001) at Time 1, but not at Time 8, *t*(109) = − 0.68, *p* = 0.499. Symptoms of negative affect were not significantly different between Time 1 (*M* = 22.75, *SD* = 8.22) and Time 8 (*M* = 21.30, *SD* = 8.62); *t*(60) = 1.38, *p* = 0.172 for female participants, nor male participants between Time 1 (*M* = 19.18, *SD* = 7.82) and Time 8 (*M* = 20.22, *SD* = 7.93); *t*(49) = − 0.776, *p* = 0.221. Female participants showed significantly higher vividness of negative future imagery (*M* = 22.84, *SD* = 5.53) compared to male participants (*M* = 19.85, *SD* = 5.85; *t*(261) = − 3.75, *p* < 0.001) at Time 1, but not at Time 8, *t*(108) = − 1.55, *p* = 0.062. Vividness of negative future imagery was not significantly different for female participants between Time 1 (*M* = 22.07, *SD* = 6.07) and Time 8 (*M* = 20.80, *SD* = 6.26); *t*(60) = 1.60, *p* = 0.116, nor male participants between Time 1 (*M* = 19.31, *SD* = 5.26) and Time 8 (*M* = 18.90, *SD* = 6.60); *t*(48) = 0.457, *p* = 0.650. There were no other significant effects of sex on any other variables,* p* > 0.124 at Time 1 or Time 8. Table 2 also shows the correlations between each variable and age. There was a significant correlation between age and negative affect at Time 1 only, *r* = 0.13, *p* = 0.034.

**Table 2:** Means and standard deviations for negative affect, anhedonia, positive and negative future imagery for the whole sample and by sex, and correlations between each variable and age

	Whole Sample Mean (SD)	Female Mean (SD)	Male Mean (SD)	Correlations with age
	N = 268	N = 129	N = 139	N = 268
T1 Negative Affect	20.90 (8.39)	23.01 (8.82)	18.94 (7.48)	.13*
	N = 111	N = 61	N = 50	N = 111
T8 Negative Affect	20.81 (2.30)	21.30 (5.20)	20.22 (7.93)	.11
	N = 286	N = 129	N = 139	N = 268
T1 Anhedonia	15.57 (5.09)	15.20 (4.96)	15.92 (5.20)	.05
	N = 111	N = 61	N = 50	N = 111
T8 Anhedonia	15.26 (5.84)	14.96 (5.65)	15.62 (6.09)	-.02
	N = 263	N = 128	N = 135	N = 263
T1 Positive Future Imagery	25.69 (5.79)	25.73 (5.76)	25.65 (5.85)	.03
	N = 110	N = 61	N = 49	N = 110
T8 Positive Future Imagery	24.55 (6.57)	23.98 (6.23)	25.24 (6.97)	-.03
	N = 263	N = 128	N = 135	N = 263
T1 Negative Future Imagery	21.13 (5.83)	22.84 (5.53)	19.85 (5.85)	0.10
	N = 110	N = 61	N = 49	N = 110
T8 Negative Future Imagery	19.95 (6.45)	20.80 (6.26)	18.90 (6.60)	.11

^*^Correlation is significant at .05

### Correlations Between Future Mental Imagery, Negative Affect and Anhedonia

To explore the relationships between the valence of future imagery and depressive symptoms at each time-point, we looked at correlations between the vividness of positive and negative future imagery and negative affect and anhedonia at Time 1 and Time 8 (Table 3). Negative future imagery was significantly positively associated with negative affect, but not anhedonia, both within and across time. In contrast, positive future imagery was significantly negatively associated with both negative affect and anhedonia within and across time.

**Table 3:** Correlations between negative affect, anhedonia, negative future imagery and positive future imagery at Time 1 and Time 8

		Time 1				Time 8			
		*Negative Affect*	*Anhedonia*	*Negative Future Imagery*	*Positive Future Imagery*	*Negative Affect*	*Anhedonia*	*Negative Future Imagery*	*Positive Future Imagery*
Time 1	*Negative Affect*	1.00							
	*Anhedonia*	.20**	1.00						
	*Negative Future Imagery*	.28**	.10	1.00					
	*Positive Future Imagery*	− .18**	− .42**	.08	1.00				
Time 8	*Negative Affect*	.47**	.33**	.21*	− .21*	1.00			
	*Anhedonia*	.15	.54**	.05	− .36**	.33**	1.00		
	*Negative Future Imagery*	.25**	.16	.44**	.02	.24*	− .13	1.00	
	*Positive Future Imagery*	− .22*	− .31**	− .09	.50**	− .29**	− .25**	.10	1.00

Within-time cross-variable; Cross-time within-variable (stability); Cross-time cross-variable

^**^Correlation is significant at .01; *Correlation is significant at .05

### Regressions to Explore Predictive Relationships Between Earlier Future Mental Imagery and Later Negative Affect and Anhedonia

Our first regression model, in which negative affect at Time 8 was the dependent variable, was significant, *F*(2, 108) = 6.23, *p* = 0.00, R^2^ = 0.10, *p* = 0.003. Here, both negative future imagery (B = 0.339, *p* = 0.010) and positive future imagery (B = − 0.357, *p* = 0.007) at Time 1 were significant predictors of negative affect at Time 8. It is worth noting that when sex and age were entered into the regression, these relationships remained significant. Our second regression model in which anhedonia at Time 8 was the dependent variable was also significant, *F*(2, 108) = 6.61, *p* = 0.00, R^2^ = 0.11, *p* = 0.002. However, while positive future imagery (B = − 0.322, *p* =  < 0.001) at Time 1 was a significant predictor of Time 8 anhedonia here, negative future imagery was not (B = 0.134, *p* = 0.141).

### Cross-Lagged Models to Simultaneously Assess Reciprocal Paths Between Future Mental Imagery, Negative Affect and Anhedonia

Figure 1 presents path coefficients for a cross-lagged panel model between negative future imagery and negative affect, controlling for sex and age at Time 1 only. Fit statistics suggested good overall fit (χ^2^ = 3.52, *p* = 0.62, CFI = 1.00, TLI = 1.00, RMSEA = 0.00 SRMR = 0.03). Significant path coefficients between repeated measures of each construct (negative future imagery_T1-T8_ = 0.48, negative affect_T1-T8_ = 0.41) suggested stability of temporal associations across time. For concurrent (i.e. within-time) associations between negative future imagery and negative affect, a significant association was only observed at Time 1 (0.20). Finally, despite significant correlations (shown in Table 3), cross-time associations between negative future imagery and negative affect were non-significant once the stability and concurrent associations were included in the model.

Figure 2 shows path coefficients for a cross-lagged model between positive future imagery and negative affect, controlling for sex and age at Time 1 only. Fit statistics suggested good overall fit (χ^2^ = 1.25, *p* = 0.98, CFI = 1.00, TLI = 1.00, RMSEA = 0.00 SRMR = 0.02). Significant path coefficients between repeated measures of positive future imagery_T1-T8_ = 0.50 and negative affect _T1-T8_ = 0.40 suggested stability of each variable across time. Concurrent associations between positive future imagery and negative affect were only significant at Time 1 (-0.19). Cross-lagged path coefficients capturing causal associations between positive future imagery and negative affect were non-significant between Time 1 and Time 8.

Figure 3 shows path coefficients for a cross-lagged model between positive future imagery and anhedonia. Model fit could not be assessed because the model was saturated. Significant path coefficients between repeated measures of positive future imagery_T1-T8_ = 0.49 and anhedonia _T1-T8_ = 0.47 suggested stability of each variable across time. Concurrent associations between positive future imagery and anhedonia were only significant at Time 1 (-0.45). Cross-lagged path coefficients capturing casual associations between positive future imagery and anhedonia were non-significant between Time 1 and Time 8.

## Discussion

This study aimed to extend previous cross-sectional associations between future mental imagery variables and negative affect and anhedonia by investigating whether i) negative and positive future imagery predicted later negative affect, and ii) whether positive future imagery predicted later anhedonia. Regression analyses showed significant associations between Time1 negative future imagery and Time 8 negative affect, Time 1 positive future imagery and Time 8 negative affect, and Time 1 positive future imagery and Time 8 anhedonia. However, using three cross-lagged panel models that controlled for within-construct longitudinal stability and cross-sectional associations between negative/positive future imagery and negative affect/anhedonia at Time 1, we did not obtain any significant cross-lagged pathways. We also confirmed at each time-point, the two-factor structure of the Prospective Imagery Task in an adolescent Israeli population.

Our first set of findings replicated previous cross-sectional results in a UK sample of young people, which suggest that negative affect and anhedonia have unique cognitive correlates [21]. These results could mean that targeting both aspects of imagery could be helpful in ameliorating two key symptoms of depression, thus adding to intervention innovation efforts. This is particularly important as current treatments of depression do not adequately treat symptoms of anhedonia, with a greater focus on alleviating negative mood than enhancing positive affect [7, 8, 10]. Our results indicate that targeting positive future mental imagery may be an important treatment target for reducing symptoms of anhedonia in adolescents, particularly as anhedonia is predictive of poorer clinical outcomes (Pizzagalli, 2022) and increased suicidal ideation, even after depressive symptoms have been controlled for (Ducasse et al., 2018). Indeed, imagery-based treatments that increase the vividness of positive future images have been shown to increase positive affect and reduce anhedonia in depression within adult populations [3, 14, 15]. These treatments are thought to increase positive affect by increasing positive imagery bias [3, 15] and increasing anticipatory pleasure [14]. Future research should investigate whether these results can be replicated in depressed adolescent populations, but potentially in other mental health problems where anhedonia is present, such as schizophrenia [31] and eating disorders [9].

Despite the promising bivariate and multivariate findings, findings from our cross-lagged panel models indicate that the effects of future mental imagery on symptoms of negative affect and anhedonia do not “carry” across time as trait-like precursory factors. Instead, these associations between future mental imagery variables and these core symptoms of depression appear to reflect state “in the moment” effects—although even this was limited to the earlier time-point. These data may suggest that associations between mental imagery cognitions and depression symptom dimensions emerge earlier in development, and account for later correlations between these variables. In turn, these data may point to the delivery of preventative skills training in mental imagery earlier, to modify later time/context-specific effects of mental imagery on negative affect and anhedonia. Unlike some resource-intensive cognitive behavioural interventions, research has shown that psychological interventions targeting mental imagery variables can be delivered in schools and reduce symptoms of depression, as well as increasing memory specificity and ability to imagery future events [33]. Future research could test early intervention (prevention) studies with long follow-up periods of the course of depression symptoms.

The Prospective Imagery Task was translated into Hebrew, and we gathered data to support its two-factor structure in a population of Israeli adolescents. This factor structure is consistent with the original factor structure of the PIT [40], as well as the factor structure demonstrated in a large UK adolescent sample [21] and a large Chinese sample of adolescents and adults [26]. We found that the psychometric properties of the Hebrew version of the PIT, in terms of internal consistency, were consistent with previous studies [21, 26, 40], suggesting that the PIT can be used to measure prospective mental imagery in both English and non-English speaking samples. To our knowledge, this is the first time the PIT has been used in an Israeli sample and our results suggest that a Hebrew version of the PIT can measure prospective mental imagery in an Israeli adolescent population. Whilst the PIT demonstrated good internal consistency, we found that the test–retest reliability of the PIT subscales was poor in this study. Previous research [26] found acceptable and good, respectively, test–retest reliability for the negative and positive subscales of the PIT, although this was over a slightly shorter duration (two months) than our study. Future studies should assess the face validity of the PIT in an Israeli sample.

We explored the effects of demographic factors on positive and negative future imagery, and negative affect and anhedonia. We found that females reported significantly greater symptoms of negative affect than males, but only at Time 1. This is consistent with previous research which found that women report more negative affect than men [13], though this was in an adult population. Additionally, we found that females reported greater vividness for negative future events than males, but again only at Time 1. This finding is broadly consistent with previous research which found that, in adolescents, females reported less vivid positive future imagery and more vivid negative future imagery than males [21]. Unexpectedly, we only found these sex differences at Time 1. Examining the means, it appears that negative affect and vividness of negative future imagery reduced for female participants over time, whereas negative affect increased for males over time, though these cross-time comparisons were non-significant for either females or males. These small, non-significant, changes may have closed the gap between females and males at Time 8, and with the lower power due to attrition any sex differences may have become non-significant.

The current study has some limitations. First, our sample size at baseline was small, and this suffered from additional attrition (41%). We therefore may not have had enough power to detect weaker cross-lagged paths. We also could not put all variables together in one overall model with both negative affect and anhedonia at Time 8 as outcome variables. Second, our results rely on self-report measures, particularly the PIT, which means we do not know whether images generated by participants are actually detailed and vivid; nor do we know of their content. Additionally, as our data was collected during the lockdown phases of COVID-19 pandemic, it is unclear how generalisable our findings are beyond the pandemic. It is possible that vividness ratings may have been influenced by life events related to events on the PIT, e.g. “You will have lots of fun with friends” may have been less vivid to a young person in lockdown during the COVID-19 pandemic. Third, it is unclear what effect repeated assessments of these measures have on their validity. Repeated assessments could contribute to participants repeating the same responses across time (similar to practice effects) or lead to boredom and disengagement with the task and therefore less valid responses. In this context, it is worth noting that the test–retest correlations for the different measures were generally low, which may have been due to repeated testing. Lastly, as our sample only consisted of young people from Israel, our results may not generalise more globally across ethnicities. Future research is needed to replicate these findings in larger and more diverse samples, as well as in contexts outside of lockdown restrictions.

In summary, our results indicate that symptoms of negative affect are associated with having more vivid negative future imagery and less vivid positive future imagery, and symptoms of anhedonia are only associated with having reduced vividness of positive future imagery, but these effects may be time-limited. These findings extend previous cross-sectional results and may add to the potential of targeting prospective mental imagery in prevention programmes for adolescent depression.

## Summary

Adolescent depression is associated with unhelpful emotional mental imagery. Cross-sectional research has shown that symptoms of negative affect are associated with more vivid negative future imagery and less vivid positive future imagery, and symptoms of anhedonia are only associated with reduced vividness of positive future imagery. However, it is unclear whether, in adolescents, depressive symptoms temporally-predict prospective mental imagery vividness or whether prospective mental imagery vividness predicts depressive symptoms. The present study therefore aimed to establish temporal relationships between these variables using longitudinal data from a sample of 111 Israeli adolescents. Using three cross-lagged panel models, at Time 1, we extended the previous cross-sectional findings between symptoms of adolescent depression (negative affect and anhedonia) and vividness of prospective positive and negative mental imagery. Despite promising bivariate and multivariate findings, once ‘concurrent’ (across-variable, within-time) and ‘stability’ paths (across-time, within-variable) were estimated, we did not find significant cross-lag paths of earlier prospective mental imagery predicting later symptoms of depression in adolescents. However, it is worth noting that attrition was high in this study, and future research should therefore consider exploring these associations in larger, more diverse populations. Extending the cross-sectional findings suggests that targeting prospective mental imagery may be important in prevention programmes for adolescent depression.
